# Addition of *Aegilops biuncialis* chromosomes 2M or 3M improves the salt tolerance of wheat in different way

**DOI:** 10.1038/s41598-020-79372-1

**Published:** 2020-12-18

**Authors:** Eva Darko, Radwan Khalil, Zsanett Dobi, Viktória Kovács, Gabriella Szalai, Tibor Janda, István Molnár

**Affiliations:** 1 Department of Plant Physiology, Centre for Agricultural Research, Agricultural Institute, Martonvásár, 2462 Hungary; 2 Botany Department, Faculty of Science, Benha University, Benha, 13518 Egypt; 3 Institute of Experimental Botany, Centre of the Region Haná for Biotechnological and Agricultural Research, 78371 Olomouc, Czech Republic; 4 Department of Plant Genetic Resources, Centre for Agricultural Research, Agricultural Institute, Martonvásár, 2462 Hungary

**Keywords:** Plant breeding, Environmental impact

## Abstract

*Aegilops biuncialis* is a promising gene source to improve salt tolerance of wheat via interspecific hybridization. In the present work, the salt stress responses of wheat-*Ae. biuncialis* addition lines were investigated during germination and in young plants to identify which *Aegilops* chromosomes can improve the salt tolerance of wheat. After salt treatments, the *Aegilops* parent and the addition lines 2M, 3M and 3M.4BS showed higher germination potential, shoot and root growth, better CO_2_ assimilation capacity and less chlorophyll degradation than the wheat parent. The *Aegilops* parent accumulated less Na in the roots due to an up-regulation of *SOS1, SOS2* and *HVP1* genes, while it contained higher amount of proline, fructose, glucose, galactose, maltose and raffinose. In the leaves, lower Na level was accompanied by high amount of proline and increased expression of *NHX2* gene. The enhanced accumulation of sugars and proline was also observed in the roots of 3M and 3M.4BS addition lines. Typical mechanism of 2M addition line was the sequestration of Na into the vacuole due to the increased expression of *HVP1* in the roots and *NHX2* in the leaves. These results suggest the *Aegilops* chromosomes 2M and 3M can improve salt tolerance of wheat in different way.

## Introduction

Wheat (*Triticum aestivum* L.) is one of the most essential cereals worldwide consumed by approximately 20% of human population. According to prognostics, wheat productivity should be doubled by year 2050^1^ in order to produce an adequate quantity of food for the escalating human population, however wheat productivity is often reduced by environmental stresses such as drought and salinity. High salinity affects around 20% of the arable land^2^. Wheat is a sensitive or moderately salt tolerant species and soil salinity above ~ 10 dS m^−1^ (or above app. ~ 100 mM NaCl in a solution) restricts plant growth and leads to significant yield loss, especially in arid and semiarid regions^2^.

Improvement of the salt tolerance of wheat has become a great challenge and important goal for plant breeders; however, it has been resolved with restricted success yet due to the complex and multigenic nature of salt tolerance^3^. The complexity is based on the fact that salt stress causes osmotic, ionic and oxidative stress in plants and the protection against them requires a complex physiological and metabolic rearrangement in the plants.

At least, three major mechanisms contributing to salinity tolerance are identified, namely restricted uptake and transport of Na in roots and shoot, Na exclusion from cytosol causing tissue tolerance and protection against salt-induced osmotic stress^4,5^.

Accumulation of compatible solutes, such as soluble sugars (e.g. fructose, glucose, galactose, raffinose and trehalose), proline, glycine betaine (GB), and polyols can serves as osmoprotectants (for review see Krasensky and Jonak^6^). Enhancement in the osmotic adjustment capacity may also improve the salt tolerance of plants^7,8^.

Other potential mechanisms responsible for salt tolerance are restricted Na^+^ uptake by the roots and/or transport to the shoot either by reduced Na loading into xylem or its recirculation into the roots (for review see Hanin et al.^3^). Several transporters were identified, which take part in these processes: SOS1 (a plasma membrane Na^+^/H^+^ exchanger) in interaction with SOS2 and SOS3 proteins^9^ operates in Na-exclusion. HKT transporters mediates Na distribution between roots and shoots by inducing Na retrieval from the shoot. It prevents the leaves from Na overaccumulation^10^. In addition, the maintenance of low cytoplasmic Na^+^ concentration can be achieved by efficient sequestration of Na to the vacuole. The tonoplast Na^+^/H^+^ antiporters (NHXs) energized by the vacuolar H^+^-ATPase and pyrophosphatases (HVPs) are found to take part in these processes. However, some of these transporters, like HKTs and NHXs, also regulate the K^+^ level in different plant tissues and play a role in the retention of K^+^ in cytosol^11,12^. The ability to retain potassium in cytosol is just as important as the elimination of sodium in order to maintain the metabolic processes. Therefore, K retention in the cells is also a good indicator of salt tolerance^13^.

All these mechanisms, together or separately, can contribute to the sustaining of normal cell activities, and therefore they may be efficient under a particular circumstance and/or in a genotype- and growth stage-dependent way^14,15^. According to Munns et al.^14^ Na^+^ exclusion may be more effective in higher salinity, while under moderately saline conditions, the ‘osmotic tolerance’ may be much more pronounced. In both cases, it is likely that salt tolerance is a results of the harmonized coordination of a vast number of genes. The main challenge in improving salt tolerant wheat cultivars is to identify those allele combinations that ensure these coordinated functions. Moreover, the variability of these allele combinations is low in domesticated wheat due to thousands of years of long cultivation and breeding^16,17^. Involvement of new, alternative gene sources in breeding programmes may enhance genetic diversity of wheat and may improve its potential for salt tolerance.

*Aegilops* species are the closest relative of wheat and represent a huge reservoir of useful gene variants that can be used in breeding programmes through interspecific hybridization^18,19^. Several wheat—*Aegilops* hybrids, addition and translocation lines have already been developed for this purpose^20^. These introgression lines have been used to assign useful alien traits to transferred chromosomes and for studying the effect of alien chromosomes on the metabolomics and gene expression of wheat^18^. The chromosome-mediated approach was most successful in case of biotic stress resistance traits as more than 40 genes providing resistance against various rust diseases were transferred from *Aegilops* species into wheat^18^. Numerous data are also available for improving grain quality related traits such as micronutrient or edible fibre content^21,22^. Rather limited information is available on the successful utilization of *Aegilops* species for increasing wheat tolerance to abiotic stresses, especially to drought or salt stress^23–25^. Although many accessions of several *Aegilops* species live in arid and semi-arid regions and show good tolerance to drought or salt stress^26,27^, their genetic potentials have not been exploited yet^28^. It is due to the fact the genetic background of abiotic stress tolerance is hardly available in the wild relatives of wheat^29^. It is especially true for the allotetraploid *Aegilops biuncialis* (2n = 4x = 28) possessing UUMM genome. Up to now, several wheat—*Ae. biuncialis* disomic addition lines have been developed carrying the chromosomes 1U, 3U, 6U, 2M, 3M, 5M and 7M besides the whole wheat genome, respectively^30–32^. In addition, a 3M.4BS translocation line possessing the 3M chromosome arm from *Ae. biuncialis* on the short arm of wheat chromosome 4B has also been reported^33^. The introgression lines are suitable for studying the transfer of salt tolerance traits into wheat and to assign tolerance traits to alien chromosomes.

In the present work, we investigated the salt tolerance of wheat—*Ae. biuncialis* chromosome addition lines together with their parental genotypes. The effect of alien chromosomes on the salt stress responses were characterized at multiple level by monitoring germination potential, growth, relative water content (RWC), photosynthetic activity and chlorophyll content of the leaves characterized by SPAD (Soil Plant Analysis Development) values. Furthermore, several salt tolerance mechanisms related to the synthesis of compatible solutes and Na^+^ and K^+^ transport processes together with the expression of same key genes were also investigated. By detailed characterization of salt stress responses we provided information to the following questions: (1) is the parental *Ae. biuncialis* accession suitable gene source to improve salt tolerance of wheat, (2) is it possible to improve salt tolerance by any of the added *Aegilops* chromosomes and which additional chromosomes are responsible for the increased salt tolerance, and finally (3) how the salt tolerance mechanisms of wheat have been modified by the added *Aegilops* chromosomes?

## Results and discussion

### Salt stress response during germination

Seed germination is the first stage of a plant’s life cycle and salt tolerance of plants at germination stage is critical for successful growth under saline conditions. Under salt stress conditions, seed germination can be reduced due to the loss of seed viability and/or to the delay of germination. The former is manifested in the decrease of germination percentage (G %) and the later in the reduction of the shoot and roots length and weight, respectively^34^.

Under control condition, the best germination potential and the longest root and shoot length were observed in 3M.4BS translocation line, while the root and especially the shoot growth was slightly reduced in the addition lines 3U and 7M in comparison to the wheat parent (Supplementary Table S2).

**Figure 1:**
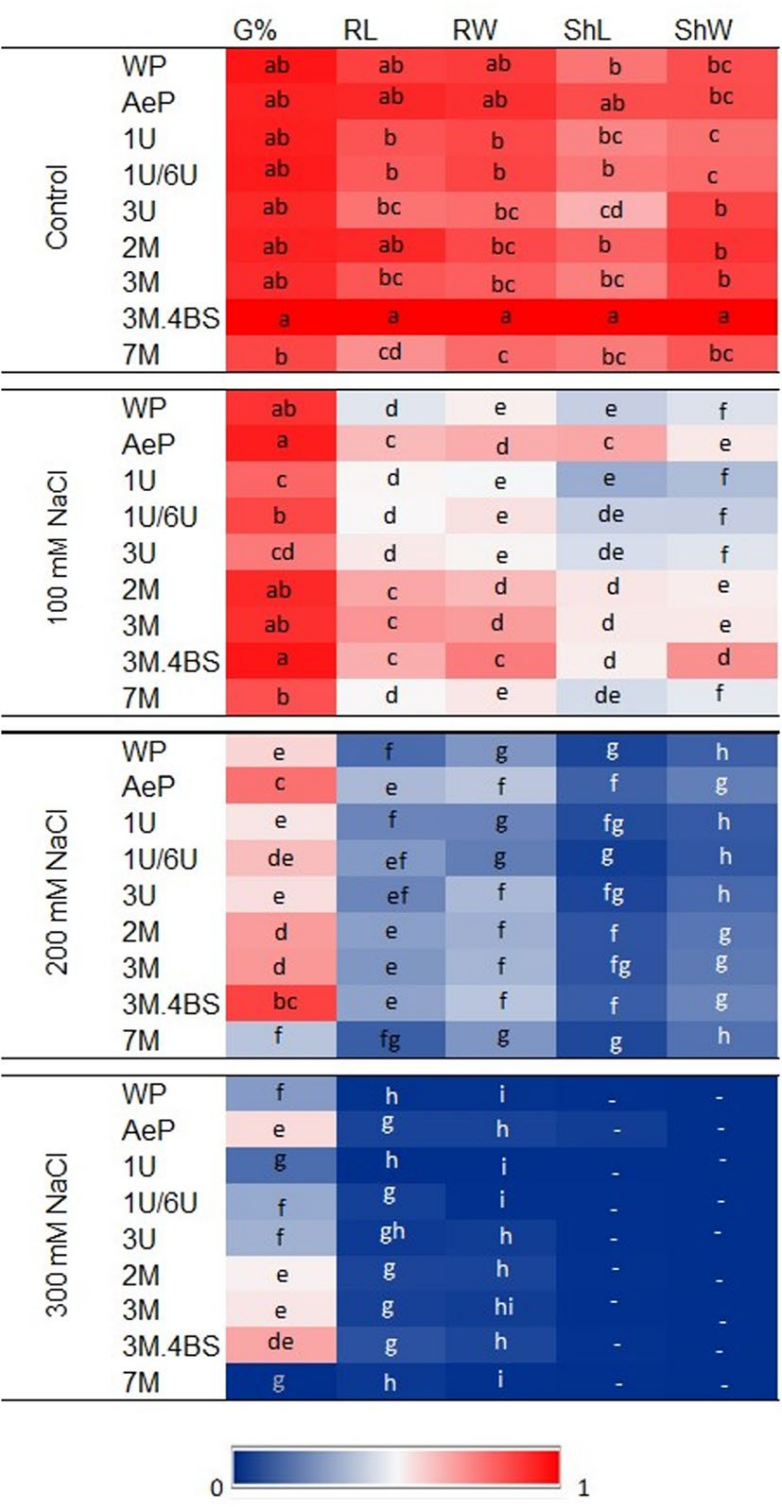
Comparison of germination properties (germination rate [G%], root length [RL], root weight [RW], shoot length [ShL], and shoot weight [ShW]) of seedlings of different addition lines and wheat (WP) and *Aegilops* (AeP) parental lines in the control (without salt treatment) and under salt stress conditions induced by 100, 200 and 300 mM NaCl treatment. Each parameter is scaled between 0 and 1 values, where dark blue represents the lowest value (0) and intense red represents the highest value (1). The absolute values are found in Supplementary Table S2.

**Figure 2:**
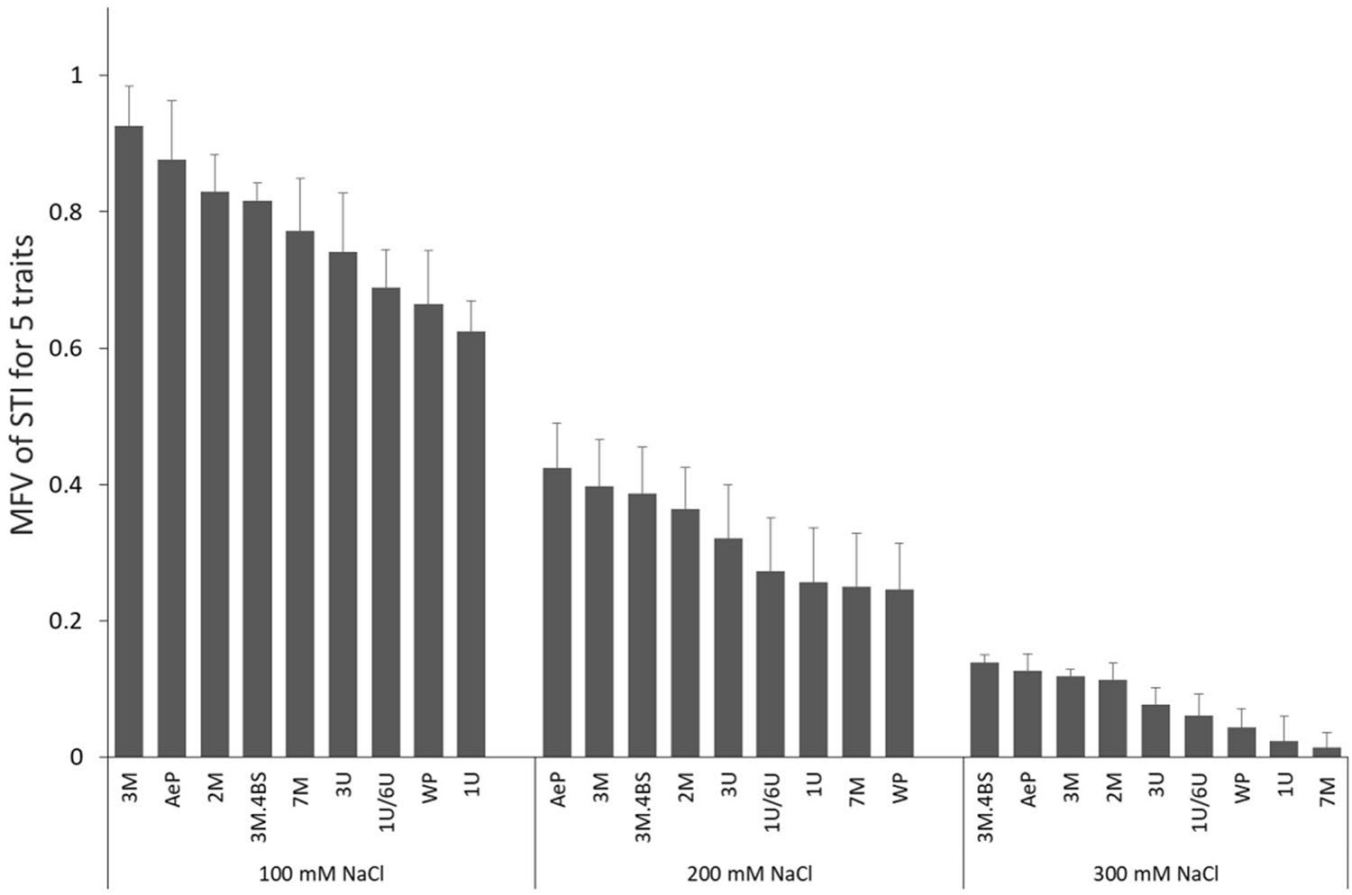
Membership functional values (MFV) of salt tolerance indices (STI) according to 5 traits of each genotype (WP–wheat parent; AeP—*Aegilops* parent and addition lines). The calculation was performed as described by Chen et al.^35^.

The salt-induced changes caused the lowest effect on G % (the reduction ranged between 0–15% at 100 mM NaCl, 20–50% at 200 mM and 30–80% at 300 mM NaCl). This was followed by the reduction of root growth, but the most pronounced effect was observed for shoot growth. The reduction of root and shoot growth ranged between 20–60% of the control’s at 100 mM NaCl and between 60 and 80% at 200 mM NaCl. At 300 mM salt concentration the shoot growth was inhibited so extensively (e.g. the shoot length ranged from 1 to 2.5 mm) that their weights were left out of consideration (Supplementary Table S2). Comparing the genotypes, however, G % was significantly higher in the *Aegilops* parent and in the wheat–*Aegilops* addition lines 2M, 3M and 3M.4BS than in the wheat parent when they were exposed to 200 or 300 mM NaCl (Fig. 1). Greater shoot and root lengths were also found in the *Aegilops* parent and in the addition lines 2M, 3M and 3M.4BS at 100, 200 and 300 mM NaCl concentrations. Similar tendencies were observed for root and shoot weight (Fig. 1 and Supplementary Table S2). For the better comparison of the genotypes and the effects of treatments several effective screening methods have been developed^35,36^. In these studies, the measured parameters (G %, the length and weight of root and shoot) were arranged and ranked according to membership function value (MFV) for each trait (Fig. 1) and the salt tolerance of the genotypes was evaluated on the basis of the salt tolerance indices (the ratio of data derived from salt-treated and control plants). The ranked list of the average values of MFV of 5 traits for the salt tolerance indices are presented in Fig. 2, which evidently demonstrated that the *Aegilops* parent and some addition lines, such as 2M, 3M, and 3M.4BS, showed better germination potential and growth under salt stress conditions than other genotypes including the wheat parental line. Further investigations were performed to confirm whether the increased salt tolerance was able to manifest in young plants too.

### Salt stress response in young plants: changes in growth, RWC, photosynthetic activity and SPAD values

**Figure 3:**
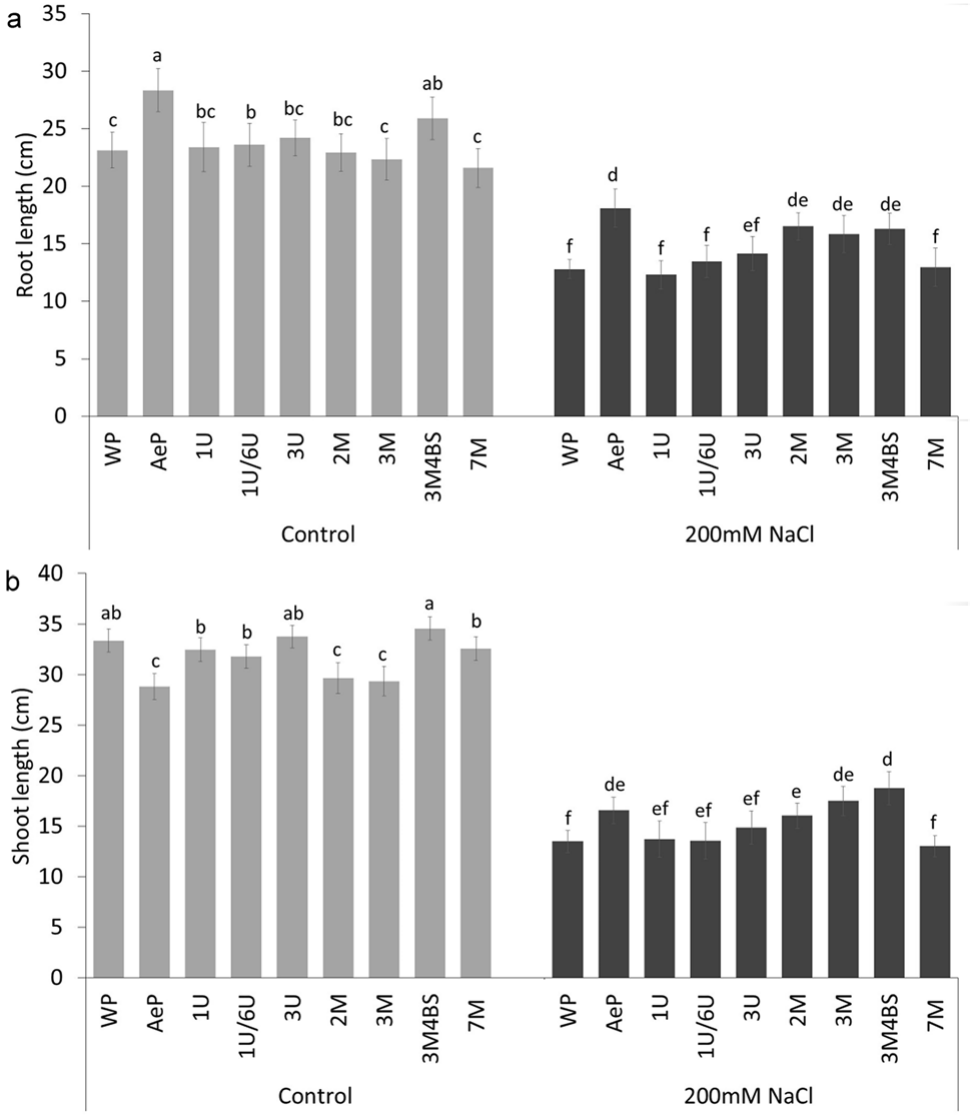
Growth responses of different genotypes grown in hydroponic solution with and without salt treatment. Root length (**a**) and shoot length (**b**) of plants. WP—wheat parent; AeP—*Aegilops* parent; Data are mean ± standard deviation of 10 replicates per treatment and genotypes. Different letters indicate significant differences between the genotypes at *p* < 0.05 using Tukey’s post hoc test.

Comparing the genotypes grown in hydroponic solution without NaCl application, the *Aegilops* accession had longer but thinner roots and shorter shoot than those of the wheat parents and most of the addition lines (except for the root of 3M.4BS translocation line and shoot of 2M and 3M addition lines) (Fig. 3). The 3M.4BS genotype developed robust roots which manifested in increased root length (Fig. 3) and weight (Supplementary Fig. S1). Several authors have indicated that root architecture affects the salt tolerance of plants as roots are directly exposed to salinity and they control the uptake and transport of water, nutrients and salts to the shoot^37,38^. In the present experiments, the length and weight of both shoots and roots were sharply reduced in all genotypes (Fig. 3) when the plants were exposed to salt stress. The reduction was lower in the *Aegilops* parent and in the addition lines 2M, 3M and 3M.4BS than that in the wheat parent and other addition lines (Fig. 3). Namely, in the *Aegilops* parent and in 2M, 3M and 3M.4BS addition lines, the reduction varied between 28–37% in root and 40–45% in shoot, while in wheat parents and in other addition lines the decrease was ranged between 40–47% and 56–60%, respectively. The greater root length of the *Aegilops* parent and the 3M.4BS addition lines and the lower salt-induced changes both in the roots and in the shoots indicated a better root and shoot growth potential under salt stress condition. In the case of 2M and 3M addition lines, better growth was manifested only under salt stress conditions. Similar trends were observed for the changes of root and shoot weight (Supplementary Fig. S1).

**Figure 4:**
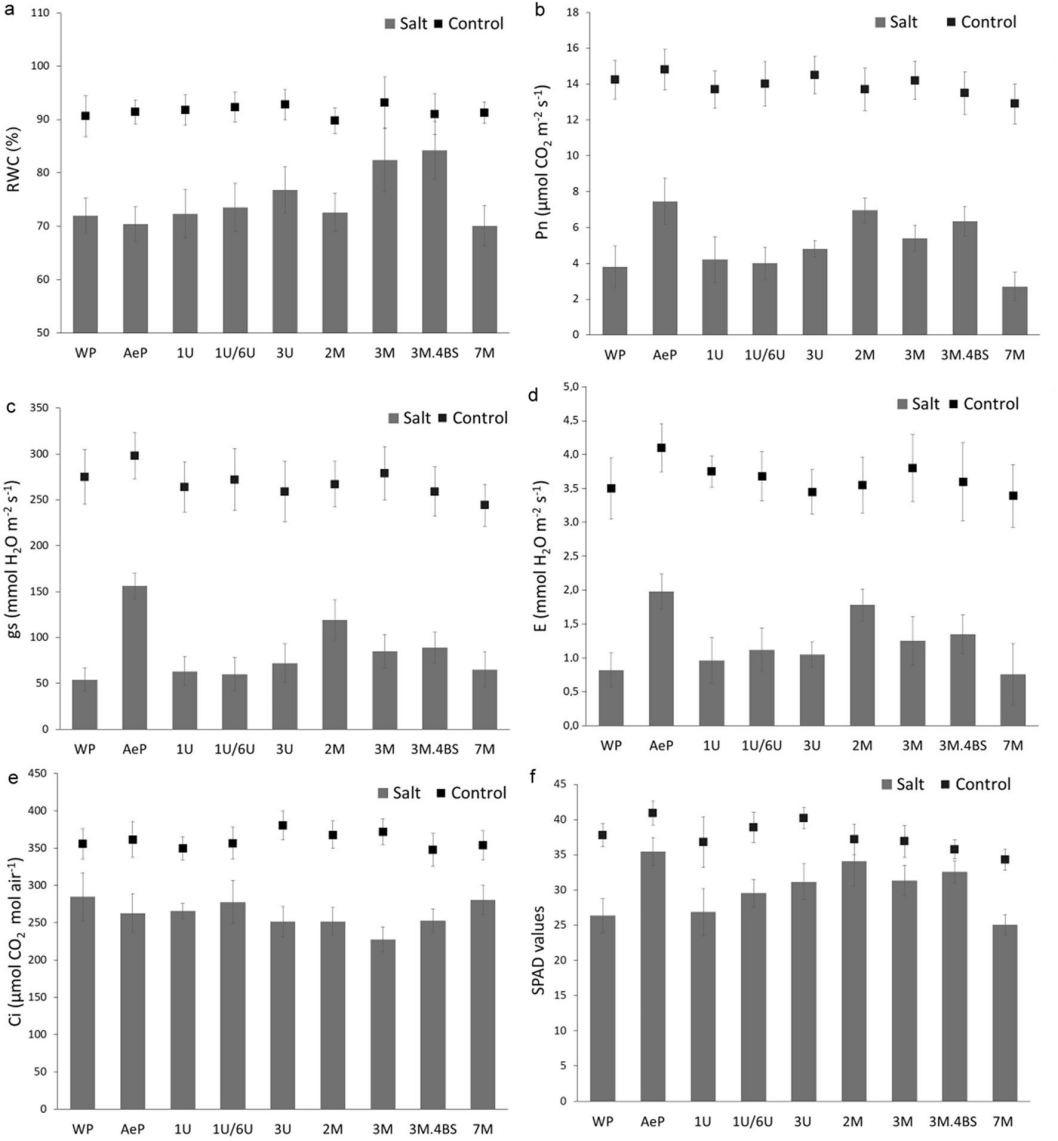
Relative water content (RWC) (**a**), photosynthetic activity (Pn) (**b**), stomatal conductance (gs) (**c**), transpiration rate (E) (**d**), interneccular CO2 level (Ci), (**e**) and chlorophyll content indicated by SPAD values (**f**) in leaves with and without salt treatment. WP: wheat parent; AeP: *Aegilops* parent. Data represent mean ± standard deviation of 5 replicates per treatment and genotype for RWC, Pn, gs, E and Ci, while n = 10 for determination of SPAD values in each treatment. The results of statistical analysis using Tukey’s post hoc test are presented in Supplementary Table S3.

Wilting of leaves is one of the most obvious symptoms of salt-induced osmotic stress, indicating a disturbance in water balance^39^. In the present study, the RWC of the leaves was similar in all plants grown in hydroponic solution without salt stress, while RWC decreased significantly under salt stress condition in most genotypes, except for 3M and 3M.4BS addition lines (Fig. 4a). It seems that these genotypes were able to maintain the water content of the leaves under salt stress.

Salinity-induced decrease in the water potential of the hydroponic solution induces stomatal closure, a decline in carbon assimilation and transpiration^40,41^. When the plants were exposed to salt stress, the decrease of CO_2_ assimilation rate (Pn), stomatal conductance (gs) related to stomatal closure and transpiration rate (E) were more pronounced in the wheat than in the *Aegilops* parent, however, no significant difference was found between the two parental lines under control conditions (Fig. 4b–d). Without salt treatment, only the 7M addition lines showed slightly lower Pn, gs and E values as compared to other genotypes. As with the *Aegilops* parent, these parameters remained higher in salt-treated 2M, 3M and 3M.4BS addition lines, indicating that these genotypes maintained their photosynthetic activity under salt stress more efficiently than others, including the wheat parent (Fig. 4b–d). The Ci level showed only a slight variation under salt stress conditions (Fig. 4e).

Chlorosis is one of the main obvious symptoms of salt stress. Chlorophyll degradation was followed by the decrease of SPAD values at the end of the experiments. Only slight differences were found in the SPAD values measured on the leaves of untreated plants, namely the 2M, 3M, 3M.4BS and especially 7M addition lines had lower values than others (Fig. 4f). Exposing of the plants to salt stress resulted in decreased SPAD values in all genotypes. However, it was less pronounced in the *Aegilops* parent and in 2M, 3M and 3M.4BS addition lines compared to other genotypes.

The measured parameters demonstrated the typical symptoms of salt stress (growth inhibition, decrease in RWC content of leaves, decline of photosynthetic activity, stomatal closure and chlorosis), however, these changes were genotype-dependent. Compared to the wheat parent, despite the wilting of leaves, the *Aegilops* parent maintained high photosynthetic activity and kept the stomata open which manifested in higher growth potential under salt stress. The addition lines 1U, 1U/6U, 3U showed similar salt stress responses to those of the wheat parent, while addition lines 2M, 3M and 3M.4BS showed better growth and photosynthetic activities and greener leaves than the wheat parent.

As similar tendencies were observed during the germination test and for plants grown in hydroponic solution, it is possible to conclude that the *Aegilops* parent and the addition lines 2M, 3M and 3M.4BS possessed higher salt tolerance than the other genotypes. In this way, we could prove that a suitable *Ae. biuncialis* accession can serve as a gene source to improve the salt tolerance of wheat and that elevated salt tolerance can be manifested in wheat background. In addition, these results indicated that chromosomes 2M and 3M may contain genes or gene regulators responsible for salt tolerance. In the next, potential mechanisms related to salt tolerance were investigated in these selected lines in order to determine how the *Aegilops* chromosomes could improve the salt tolerance of plants.

### Salt stress responses in selected plants: studies on osmotic adjustment and Na accumulation

Salinity tolerance can be attributed to multiple mechanisms including accumulation of osmoprotectants, modification of Na uptake and transport and detoxification of harmful elements. To study the role of osmoregulation, several osmoprotectors such as proline, GB and sugars, were investigated in these experiments.

**Figure 5:**
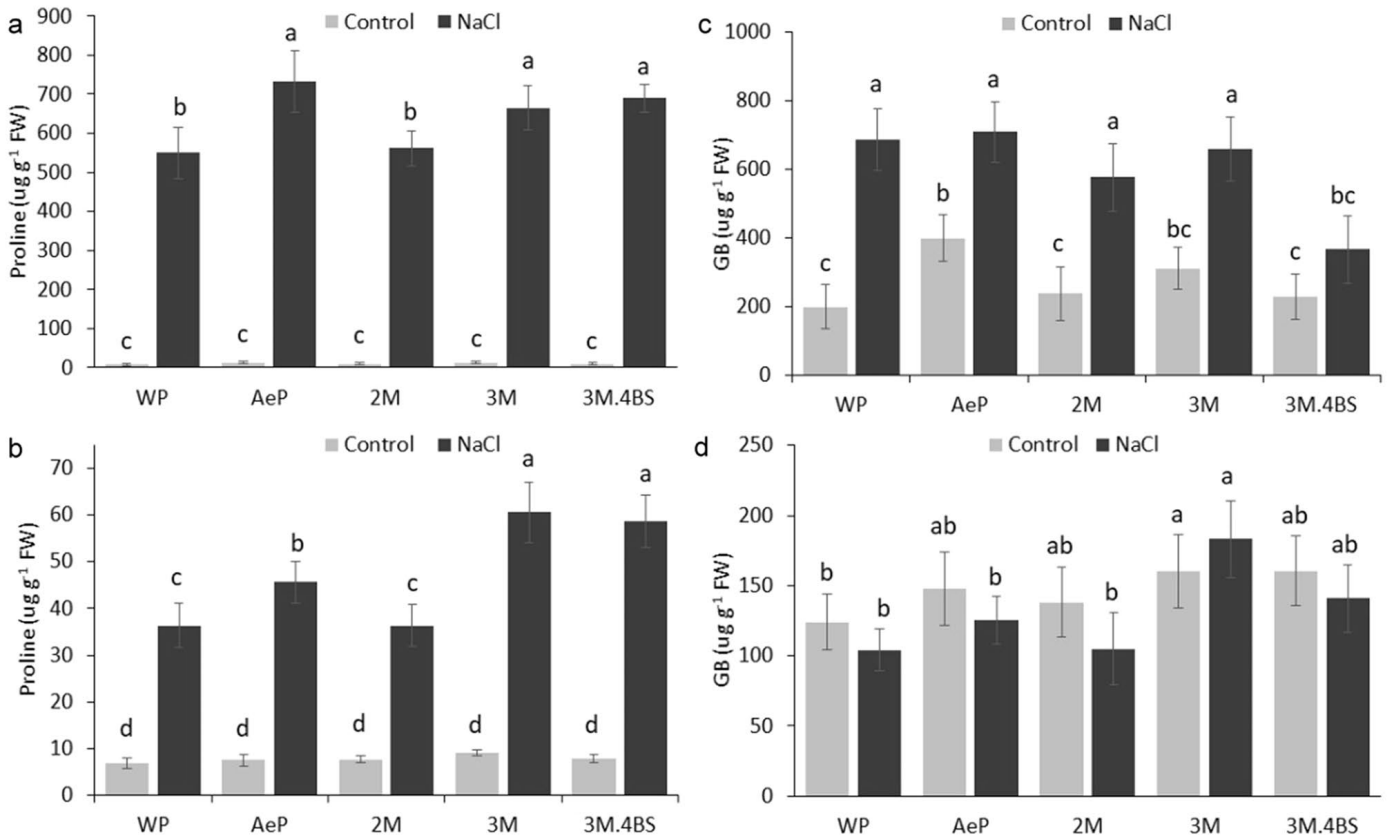
Effect of salinity treatment on proline (**a**, **b**) and glycine betaine (GB) (**c**, **d**) content of leaves (*upper panels*) and root (*bottom panels*) of different genotypes. Data are mean ± standard deviation of five replicates per treatment. Different letters indicate significant differences between the genotypes at *p* < 0.05 using Tukey’s post hoc test.

Similarly, as observed previously^42,43^, salt treatments resulted in proline accumulation both in the roots and in the leaves. Higher amount of proline was detected in the *Aegilops* parent and 3M and 3M.4BS addition lines both in the roots and the leaves (Fig. 5a,b). As concluded by Hayat et al.^44^, increased proline accumulation may result in an improved osmotic stress tolerance. While proline can help maintaining cell turgidity, they also play a role in the stabilization of macromolecules, strengthening membrane integrity, having intermediate products for carbon and nitrogen metabolism or energy production or as a ROS scavenger^45^. Although we don’t know yet what kind of mechanisms operates in these lines, it is presumed that proline can contribute to the improved salt tolerance in the *Aegilops* parent and in the 3M and 3M.4BS genotypes.

GB is also an extensively studied compatible solute. GB content did not change significantly in the roots in any lines, while it increased significantly in the leaves of all genotypes under salt stress condition (Fig. 5c,d). However, significant difference between the genotypes was found only in the case of 3M.4BS translocation line, in which the amount of GB was lower than in others. These results suggest that GB plays minor role in the salt tolerance of these genotypes.

**Figure 6:**
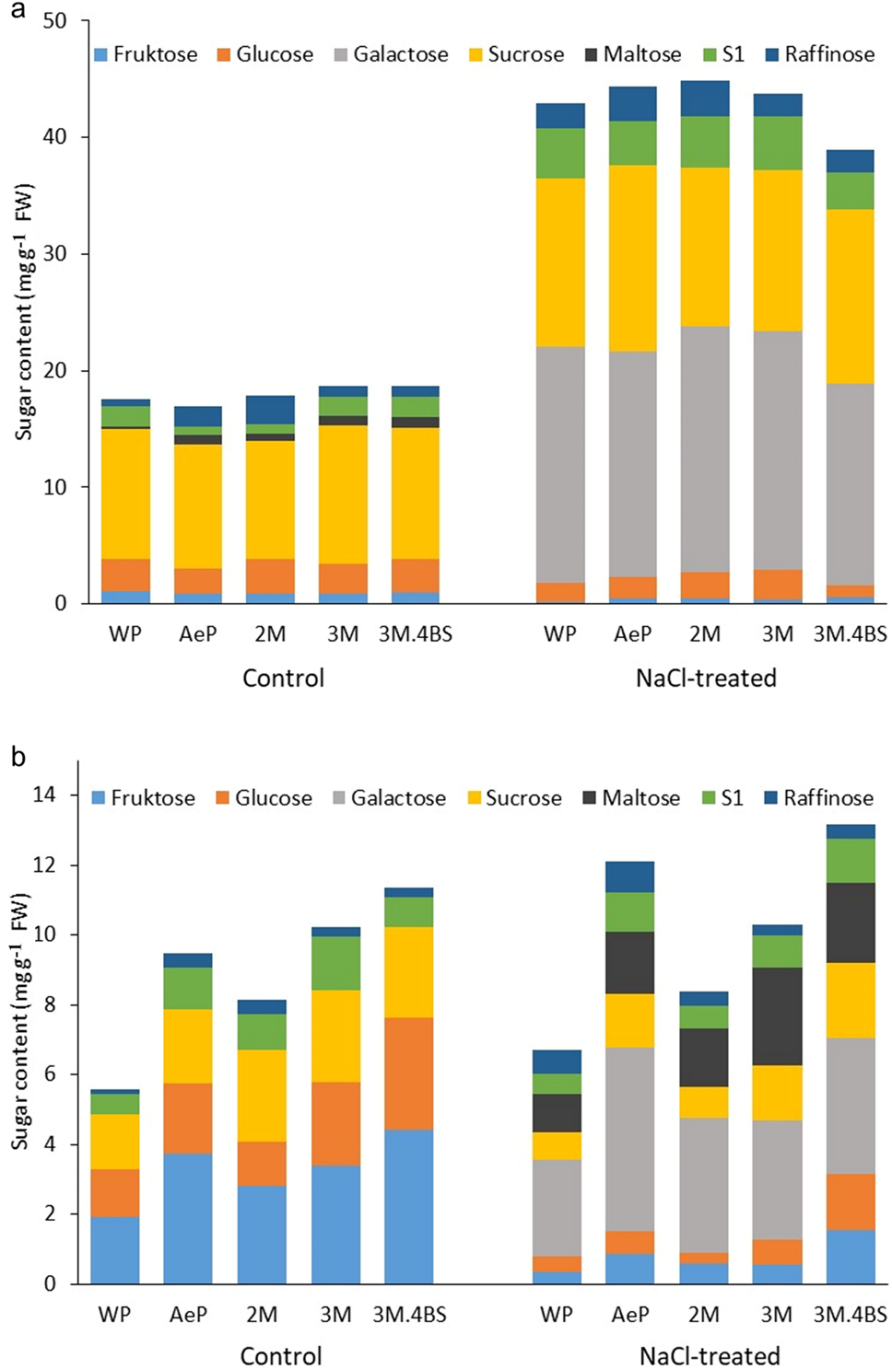
Sugar composition in the leaves (**a**) and roots (**b**) of different genotypes grown in hydroponic solution with and without salt treatment. WP–wheat parent; AeP—*Aegilops* parent and addition lines. Data are mean ± standard deviation of five replicates per treatment. Values and the results of statistical analysis using Tukey’s post hoc test are presented in Supplementary Table S4. Data are mean ± standard deviation of five replicates per treatment.

According to Al-Thani and Yasseen^46^, sugars can contribute to 30–50% of the osmotic adjustment in glycophyte plants (such as wheat). In the present study, when the plants were exposed to salt stress, the total amount of soluble sugars increased slightly (1–1.5 ×) in the roots, but intensively (2.5–3 ×) in the leaves. (Fig. 6, Supplementary Table S4). However, the sugar composition changed in both tissues. The amount of glucose and fructose decreased both in the roots and in the leaves. The sucrose content did not change or slightly increased in the leaves while it decreased in the roots. The most pronounced effect of salt treatment manifested in the accumulation of galactose both in the roots and in the leaves and also in the accumulation of maltose in the roots and that of raffinose in the leaves (Fig. 6). Starch hydrolysis, galactose or raffinose accumulation have also been detected previously in wheat under drought and different osmotic stress conditions^47,48^. In this experiment, an unidentified sugar compound (S1) was also separated between the maltose and raffinose. Its amount was high in the roots, but decreased under salt stress condition. Inversely, its amount was low in the leaves, but increased under salt stress condition. These results indicate that different metabolic pathways are activated in the roots and leaves under salt stress condition.

The above described tendencies in the sugar metabolism were observed in most of the lines; however, the comparison of the genotypes showed that the total amount of sugars were higher in the roots of the *Aegilops* parent, 2M, 3M and 3M.4BS genotypes than that of the wheat parent even without salt treatment (Fig. 6). The increased amount of sugars was maintained under salt stress condition in most of them except for 2M addition lines. The differences were mainly due to elevated amount of fructose, sucrose, S1 and raffinose in the roots of control plants, and to the higher accumulation of galactose and maltose under salt stress condition (Fig. 6, Supplementary Table S4). In the leaves, the amount and composition of sugars were similar in all genotypes under both control and salt stress conditions, only a slightly higher raffinose content was detected in the *Aegilops* parent and in 2M addition lines as compared to other genotypes.

The sugars in raffinose family oligosaccharides (RFOs) may accumulate in plant tissues during stresses, and they play a role in the development of stress tolerance^3^. During their synthesis, galactose units are transferred to sucrose^49^. Present results also confirm that sugar metabolites play important role in the osmoregulation both in the leaves and in the roots. Furthermore RFOs may play special role in the 2M additional line. According to these results, it is possible that the *Aegilops* chromosome 2M carries genes affecting the raffinose metabolism in the leaves and the chromosome 3M induces the sugar metabolism in the roots. Both of them may contribute to the altered salt tolerance of these lines through osmoprotection against salt stress. However, further investigations are necessary to determine which genes are located on them.

Salt tolerance mechanisms are also related to the modification of Na^+^ uptake and transport and/or sequestration of Na^+^ from the cytosol to the vacuoles^3^. The modification of these transport processes can also improve salt tolerance. Therefore, we examined these processes by determination of Na and K contents in the roots and leaves and of the expression of genes coding transporters taking part in Na^+^ or K^+^ transports.

**Figure 7:**
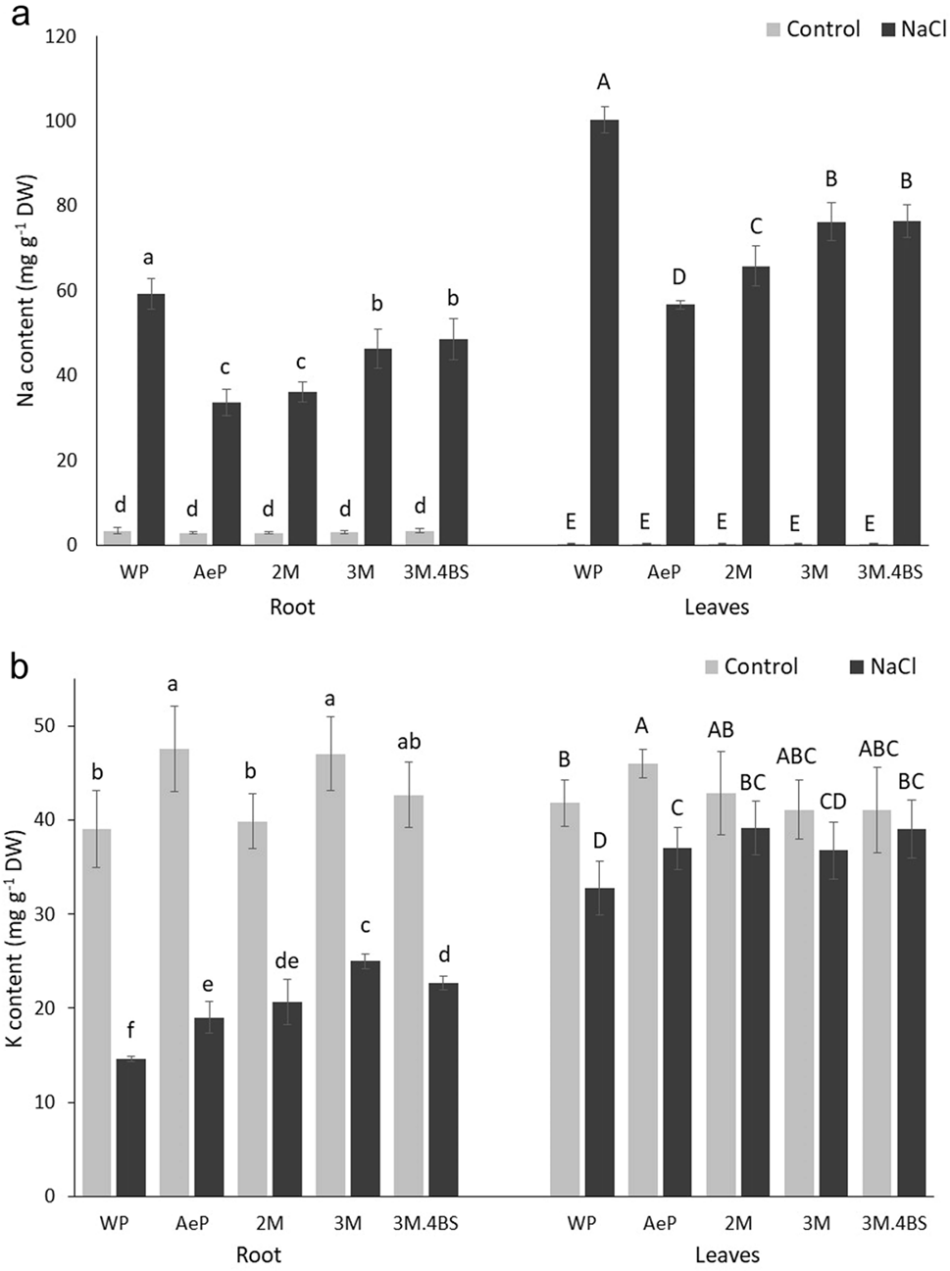
Na (**a**) and K (**b**) contents of roots (*left*) and leaves (*right*) of different genotypes grown in hydroponic solution with and without NaCl. Data are mean ± standard deviation of three replicates per treatment. Different letters indicate significant differences between the genotypes at *p* < 0.05 using Tukey’s post hoc test.

The *Aegilops* parent accumulated app. 45% less Na both in the leaves and the roots than the wheat parent (Fig. 7). Na content in the roots of 2M addition line was similar to that of in the *Aegilops* parent but in the leaves, it ranged between the two parents. In 3M and 3M.4BS addition lines, the Na content in the leaves and roots were lower than that of wheat parent and higher than that of *Aegilops* parent, however, more reduction was found in the leaves and lesser in the roots. These results indicate that both added chromosomes 2M and 3M can modify the Na^+^ transport processes but with different efficiency: the 2M chromosome caused stronger effects than 3M. On the whole, the restricted accumulation of Na either in the root or in the leaves was also accompanied with the retention of K in both tissues, as indicated by the elevated amount of K in *Aegilops* parent and addition lines, as compared to the wheat parent (Fig. 7). The similarities of the stress responses in 3M and 3M.4BS addition lines indicate that the genes responsible for the altered Na transport processes are present in the common chromosome part of 3M and 3M.4BS addition lines. Investigations are currently in progress to determine whether the short or long arm of chromosome 3M has been transferred into the 3M.4BS addition line.

**Figure 8:**
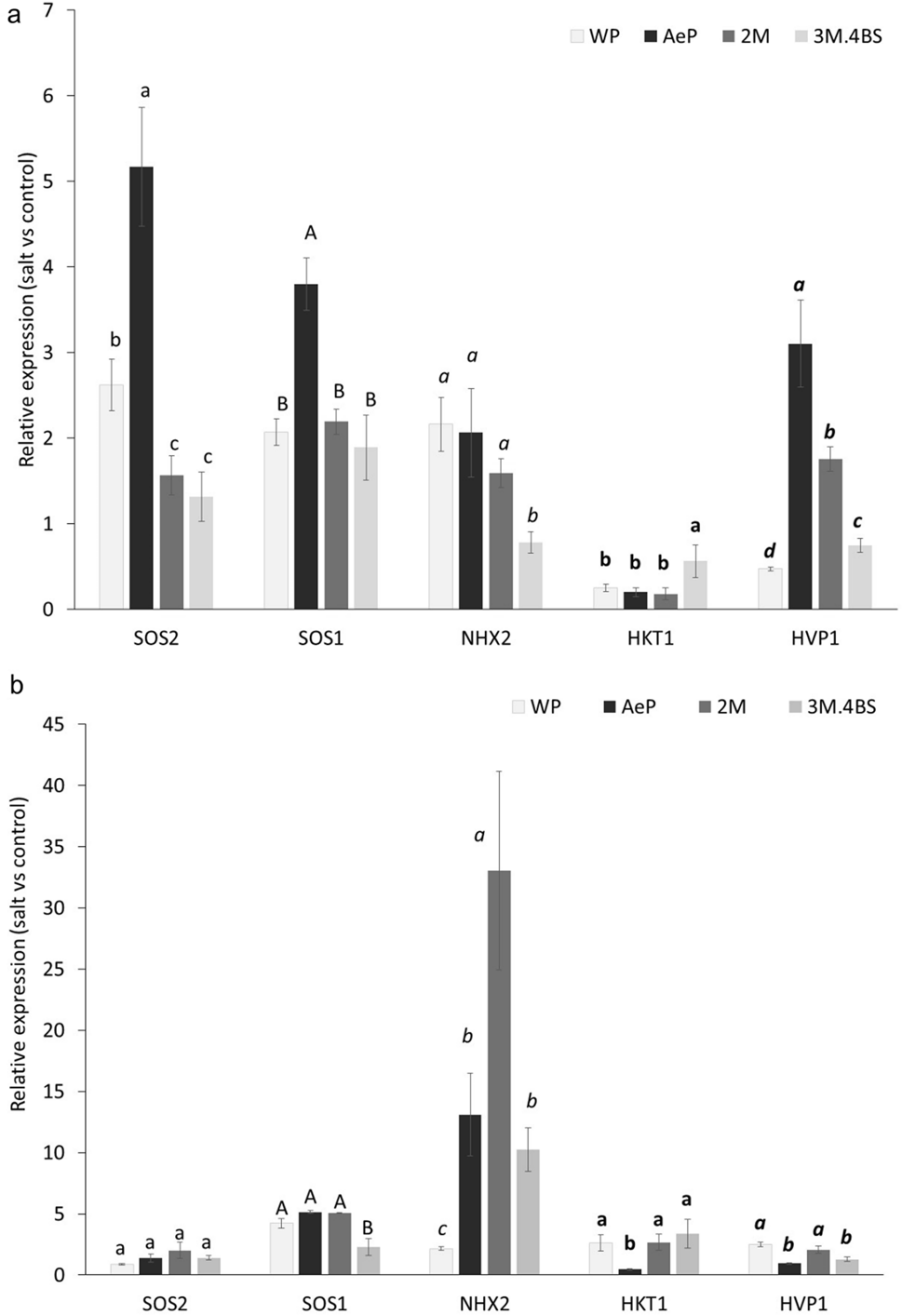
Salt-induced changes in the expression levels of the *SOS1* (*salt-overly sensitive 1*), *SOS2 (salt-overly sensitive 2*), *NHX2* (Na^+^/H^+^ antiporter), *HVP1 (vacuolar H*^+^ *inorganic pyrophosphatase 1)* and *HKT1* (histidine-kinase transporter) genes in the roots (**a**) and leaves (**b**) of wheat (WP) and *Aegilops* (AeP) parents, addition lines 2M and 3M.4BS. The data were obtained from three biological and three technical replicates of each treatment. For each gene, different letters indicate statistically significant differences between the genotypes at *p* < 0.05 using Tukey’s post hoc test.

When the expression of salt-responsive genes *SOS1, SOS2, HKT1, NHX2* and *HVP1* were investigated, the *Aegilops* parent exhibited elevated expression of *SOS1, SOS2* and *HVP1* genes in the roots and that of *NHX2* in the leaves (Fig. 8). These results are in accordance to the lower Na accumulation both in the root and in the shoots. In addition, they also indicate a higher sequestration of Na into the vacuoles. In agreement with this, Sathee et al.^50^ found increased transcript levels of *SOS1, SOS2, NHX1* and *HVP1* genes in a salt tolerant wheat genotype compared to a sensitive one, and correlation was found between the transcript level of these genes and the accumulation of Na in the roots and leaves. Furthermore, when the salt tolerance mechanism was studied in an *Ae. cylindrica* accession (US26L) originated from Uremia Salt Lake shores Norwest Iran, the *SOS1* and *HKT1* genes were strongly up-regulated in the root which correlated to the excessive exclusion of Na from the root in this salt tolerant genotype^29^.

From among the genes showing high transcript level in the *Ae. biuncialis* parent, the increased expression of *HVP1* gene in the roots and *NHX2* gene in the leaves were also observed in 2M addition line. The *NHX2* was also slightly up-regulated in the leaves of 3M.4BS line. These genes are found to be responsible for sequestration of Na to the vacuole and/or retention of K in cytosol^11^. The importance of these genes in salt tolerance was demonstrated in transgenic *Arabidopsis* and tomato plants, where the overexpression of *NHX* and *HVP* genes resulted in elevated salt tolerance^51,52^. Our results showed that the elevated expression of these genes can also be reached through chromosome-mediated gene transfer, which could be an alternative to GMO for improving the salt tolerance of wheat. Since the genome of *Ae. biuncialis* (UUMM) has not been sequenced yet, further investigations are necessary to determine which genes are located on chromosomes and 3M and/or how 2M and 3M chromosomes affect the expression of these genes.

## Conclusion

Comparing the salt stress responses of wheat and *Aegilops* parents and their addition lines 1U, 1U/6U, 3U, 2M, 3M and 7M, it was revealed that the *Aegilops* parent and the addition lines 2M, 3M and 3M.4BS possessed elevated salt tolerance both during germination and at early developmental stages. Detailed studies showed that several salt tolerance mechanisms including osmotic tolerance, Na exclusion and tissue tolerance operates more effectively in the *Ae. biuncialis* accession than in the wheat. The elevated accumulation of proline both in the roots and leaves and that of sugars, including fructose, glucose, galactose, and raffinose, in the roots suggest that these compounds can contribute to the increased osmotic protection against salt stress. Since the amount of proline and sugars were also higher in 3M and 3M.4BS addition lines, it seems that 3M chromosomes may improve the osmoregulation efficiency of wheat in the adequate tissues. Furthermore, the *Aegilops* parent accumulated less Na both in the roots and leaves due to the increased transcript level of the *SOS1, SOS2* and *HVP1* genes in the root and that of *NHX2* in the leaves. The high expression of *HVP1* and *NHX2* genes were also manifested in the 2M addition lines contributing to its improved salt tolerance, however, these processes are less important in 3M.4BS line. These investigations demonstrated that a suitable *Ae. biuncialis* accessions could be used as gene source to improve the salt tolerance of wheat and the increased salt tolerance could be transferred to wheat. The 2M and 3M chromosomes may contain genes responsible for the improved traits. From among the potential mechanisms, greater osmoprotection related to sugar and proline accumulation could contribute to the enhanced salt tolerance in those addition lines which contain 3M chromosome of *Ae. biuncialis*. The sequestration of Na into the vacuole could be the dominant mechanism in 2M addition line. These results confirmed that salt tolerance could be developed by several processes and that the enhancement of these processes, individually or together, could contribute to the improvement of such a complex trait like salt tolerance.

## Methods

### Plant materials, growth conditions and salt treatment

A partial set of wheat—*Ae. biuncialis* addition (1U, 1U/6U, 3U, 2M, 3M and 7M) and translocation (3M.4BS) lines developed from bread wheat (*T. aestivum* ‘Mv9kr1′) and *Ae. biuncialis* ‘MvGB642′ hybrid^30,33,53^ were used in these experiments together with their parental genotypes. The genetic stability of the addition lines was checked cytologically several times.

The salt response was studied during germination and in young plants as described earlier^54,55^. Briefly, in the germination test, seeds (3 × 25 of each genotype per treatment) were surface-sterilized in 1% sodium hypochlorite for 5 min, rinsed twice in distilled water and germinated on wet filter paper containing 0, 100, 200 and 300 mM NaCl, in Petri dishes for 3 days, at room temperature. Afterwards, G %, length and weight of root and coleoptile (n = 3 × 10) were determined.

For testing the salt responses in young plants, the surface-sterilized seeds were germinated on wet filter paper in Petri dishes for 3 days at room temperature. The seedlings with similar root length were grown in pots (10 plants/0.6 L pot) containing half-strength Hoagland solution in a phytotron growth chamber (PGR15, Conviron, Controlled Environments Ltd, Winnipeg, MB, Canada), under a 16 h photoperiod at 250 µmol m^−2^ s^−1^ and 22/20 °C day/night temperature for 7 days. The solutions were changed every 2 days. The 10-day old plants were treated with salt by adding 100 mM NaCl to the nutrient solution, while the control plants continued to growth in half-strength Hoagland solution. After 7 days, the NaCl concentration was increased to 200 mM for further 7 days. Five pots of each line were used as controls (without salt treatment) and five of them were used for the salt treatment. The salt-induced changes were carried out at the end of the experiments.

### Monitoring salt-induced changes in growth, RWC, photosynthetic activity and chlorophyll content of leaves

The salt-induced decrease in growth was determined by measuring the length and weight (g/plant) of the roots and shoots. At least 15 plants (3 from each pot) of each line and treatment were measured.

RWC content of leaves was determined using the following equation: RWC = (FW-DW)/(SW-DW) × 100 where FW is fresh weight, SW is water-saturated weight measured after 24 h rehydration of leaves in distilled water and DW is dried weight measured after oven drying for 48 h at 80 °C. Approximately 0.2 g of leaves were sliced into 2 cm segments for each sample and five samples were used for each treatment and genotype.

Photosynthetic activity of the plants was determined by gas exchange analysis. It was performed on the youngest fully expanded leaves using a Ciras 3 Portable Photosynthesis System with a narrow (2.5 cm^2^) leaf cuvette holder (PP, Systems Company, Amesbury, USA). Net photosynthetic rate (Pn), stomatal conductance (gs), transpiration rate (E) and intracellular CO_2_ concentration (Ci) were determined at the steady-state level of photosynthesis using a CO_2_ level of 390 μL L^−1^ and light intensity of 450 μmol m^-2^ s^−1^ . At least five leaves were measured for each line and treatment.

The chlorophyll content of the leaves was characterized by SPAD values determined with a SPAD-502 Chlorophyll Meter (Spectrum Technologies, Painfiled, IL, USA). At least 10 leaves were measured for each line and treatment.

### Determination of metabolites related to osmotic adjustment

The salt induced changes of several osmolytes were followed by the determination of the amount of proline, GB and sugar contents of the leaves and roots.

Proline and GB contents were measured photometrically using a UV–Vis spectrophotometer (160A, Shimadzu Corp, Kyoto, Japan) as described by Bates et al. (1973)^56^ and Grieve and Grattan^57^, respectively.

Sugar content, including the amount of glucose, fructose, sucrose, galactose, sacharose, raffinose and maltose, were measured after HPLC analysis performed according to Gondor et al.^58^. Standards purchased from Sigma-Aldrich (Darmstadt, Germany) were used for the quantification.

For each method, 5 × 0.3 g of leaf or root samples were collected per treatment and genotype.

### Determination of Na and K content in leaf and root

The amounts of Na and K were measured from air-dried samples (0.5 g per sample) using the inductively coupled plasma-atomic emission spectrometry method (ICP-AES, Jobin–Yvon Ultima 2 Sequential Instrument) after microwave Teflon bomb digestion with cc. HNO_3_ + HCl^59^. Three samples of each genotype and treatment were collected for analysis at the end of the experiments.

### Gene expression studies

Expression of *SOS1, SOS2, HKT1, NHX2* and *HVP1* genes responsible for Na^+^ transport and sequestration were determined by quantitative reverse transcription PCR (qRT-PCR). The RNA isolation process, the method of cDNA synthesis and the qRT-PCR measurements were performed as it was described by Darko et al.^54^. Briefly, the total RNA content was isolated from fully developed leaf and root samples with a Direct-zolRNA MiniPrep Kit (Zymo Research, USA) and TRI-Reagent (Zymo Research, USA) according to the manufacturer's instructions. M-MLV-Reverse transcriptase (Promega Corporation, Madison, WI, USA) was used for the cDNA synthesis. Quantitative RT PCR measurements were performed with the CFX96 Touch Real-Time PCR Detection System (Bio-Rad Hungary Ltd., Hungary) using the KAPA SYBR FAST, Master Mix (2X), Universal qPCR Kit (Kapa Biosystems, Inc., Wilmington, USA). Primers for the above genes were either designed with the NCBI—Primer Design Tool software (National Center for Biotechnology Information, Bethesda, USA) or the sequences were taken from the literature (Supplementary Table S1). Relative transcript levels were calculated according to the ΔΔCt method as described by Livak and Schmittgen^60^, and Ta30797 gene was used as reference. The measurements were performed using three biological and three technical replicates of each genotype and treatment.

### Data analysis and statistics

The results were obtained from three independent experiments involving 3 and 5 replicates of each genotype per treatment in the germination test and during the determination of salinity tolerance in young plants, respectively. The measurements were performed in several biological replicates as indicated above. The values presented in the figures and tables are mean ± standard deviation (SD). Tukey’s post hoc test (SPSS 16.0) was also used for determination the differences between genotypes and treatments at 0.05 significance level.

## Supplementary Information

Supplementary Information.

Supplementary Information.
