# Supplementary figures and images for: Addition of Aegilops biuncialis chromosomes 2M or 3M improves the salt tolerance of wheat in different way
Source: Sci Rep. 2020 Dec 18;10:22327. doi: 10.1038/s41598-020-79372-1 (PMC7749180; doi:10.1038/s41598-020-79372-1)

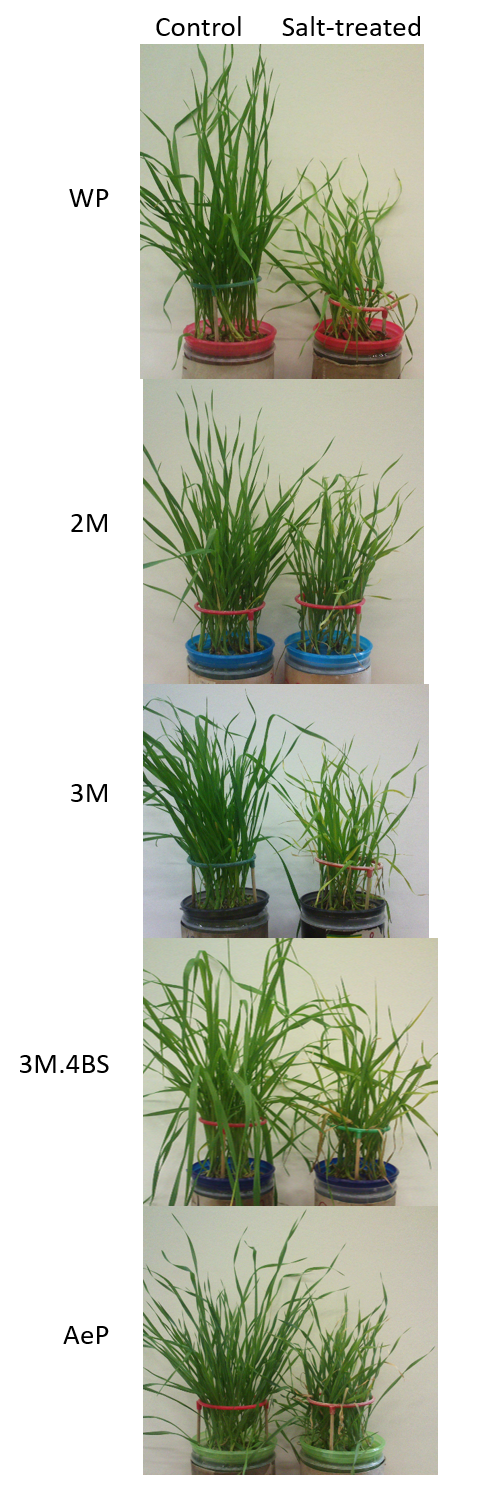

Supplement: Supplementary file 1 — Supplementary Information. [file 41598_2020_79372_MOESM1_ESM.tif]
