# Supplementary material for: Addition of Aegilops biuncialis chromosomes 2M or 3M improves the salt tolerance of wheat in different way
Source: Sci Rep. 2020 Dec 18;10:22327. doi: 10.1038/s41598-020-79372-1 (PMC7749180; doi:10.1038/s41598-020-79372-1)
Supplement: Supplementary file 2 — Supplementary Information. [file 41598_2020_79372_MOESM2_ESM.pdf]

ORIGINAL ARTICLE

Addition of *Aegilops biuncialis* chromosomes 2M or 3M improves the salt tolerance of wheat in different way

Eva Darko[1*], Radwan Khalil[2], Zsanett Dobi[1], Viktória Kovács[1], Gabriella Szalai[1], Tibor Janda[1] and István Molnár[3,4]

[1]Department of Plant Physiology, Agricultural Institute, Centre for Agricultural Research, H-2462, Martonvásár

[2]Botany Department, Faculty of Science, Benha University, Benha, 13518, Egypt

[3]Institute of Experimental Botany, Centre of the Region Haná for Biotechnological and Agricultural Research, CZ-78371 Olomouc, Czech Republic

[4]Department of Plant Genetic Resources, Agricultural Institute, Centre for Agricultural Research, H-2462, Martonvásár

*Corresponding author: Eva Darko, darko.eva@agrar.mta.hu or darko.eva@atk.hu

Running title: Salt tolerance in wheat-*Aegilops biuncialis* addition lines

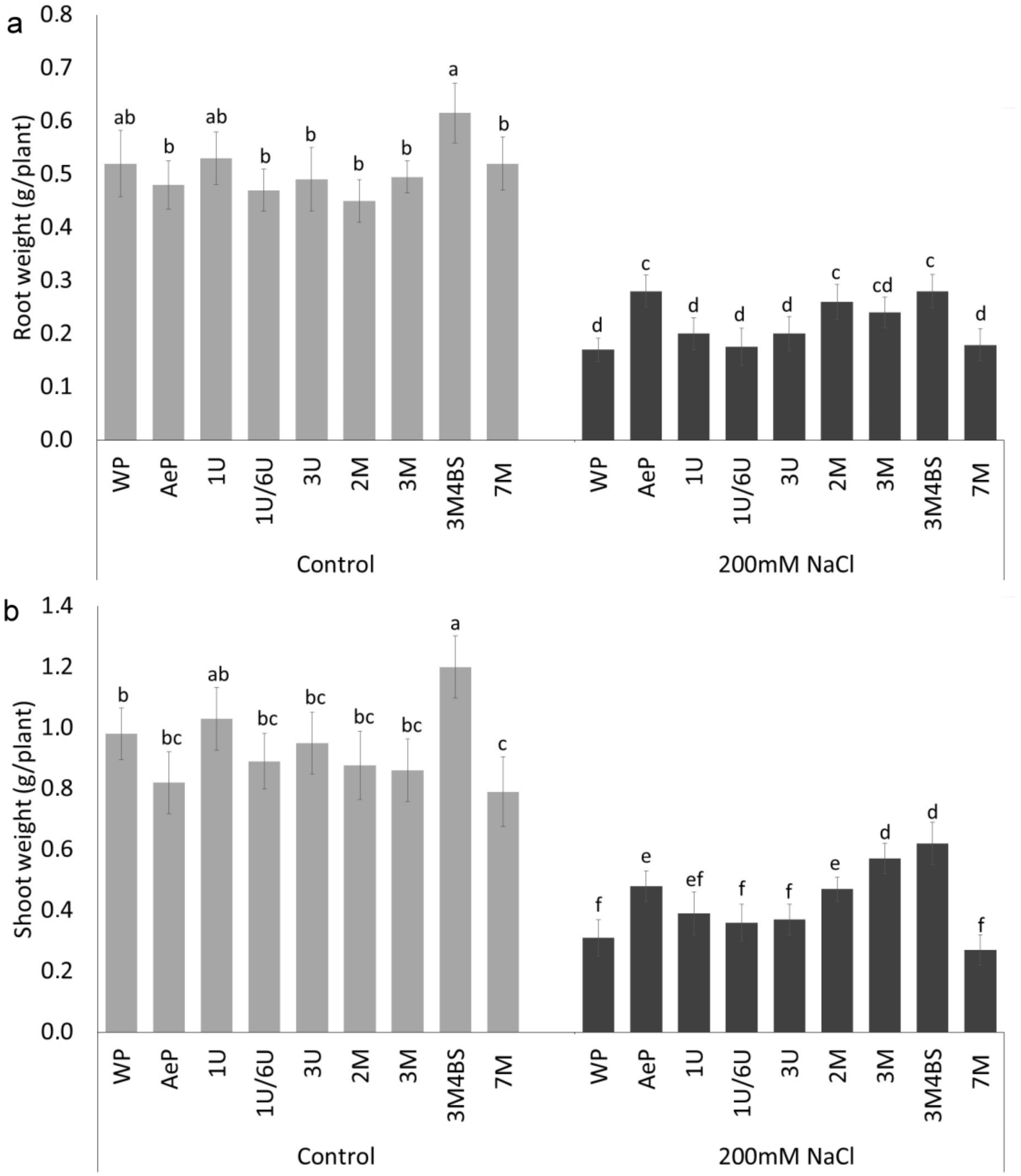

Supplementary Figure S1: Root (a) and shoot weight (b) of different genotypes grown in hydroponic solution with and without salt treatment. WP – wheat parent; AeP – *Aegilops* parent; Data are mean ± standard deviation of 10 replicates per treatment and genotypes. Different letters indicate significant differences between the genotypes at $p < 0.05$ using Tukey's *post hoc* test.

Supplementary Table S1: List of gene specific primers used in the experiments.

Name of gene		Primer sequences (5' → 3')	Gene ID	Reference
Ta 30797 (*Similar to phosphogluconate dehydrogenase*)	Forward	GCCGTGTCCATGCCAGTG	TC279294 TC284282	Paolacci et al. 2009
	Reverse	TTAGCCTGAACCACCTGTGC		
SOS1 (*Salt overly sensitive 1*)	Forward	ACACGCGGCCTCTGCTCT	KJ563230.1	Darko et al. 2017
	Reverse	CATGCTGGGAGAGTCCACT		
SOS2 (*Salt overly sensitive 2*)	Forward	GAAAACCTGCTTCTTGATTCACG	JP218831.1	Darko et al. 2017
	Reverse	GCTGCAGATCCATCATAGCC		
HKT1 (*High-affinity K^+ transporter*)	Forward	GTTGCCTGCATCACGGAAAG	U16709.1	own design
	Reverse	CTTTCCTCCGTCACTCCACC		
NHX2 (*Na^+/H^+ antiporter*)	Forward	AATAAGCTGGAGGCAGCAAA	AY040246.2	Darko et al. 2017
	Reverse	GTGCTAAACAGAACGACAGT		
HVP1 (*Vacuolar H^+-pyrophosphatase*)	Forward	TCCAAGGTCAAGGTCGCC	EU255237.1	own design
	Reverse	GGAAGACGAAGATCAGGACCG		

Supplementary Table S2: Effect of salt stress on germination properties of seedlings of wheat/*Aegilops* introgression lines and on their wheat (WP) and *Ae. biuncialis* (AeP) parents. The data are presented the mean values ± standard deviation of each treatment. The significant differences were determined using Tukey's post hoc test and the different letters indicate statistically significant difference between the genotypes and treatments at $p < 0.05$ level. The character "–" indicates that the salt treatment caused so drastic reduction in the parameter that the results of the statistical analyses, and thus the comparison of the genotypes was irrelevant due to the really low values of the data.

		WP	1U	1U/6U	3U	2M	3M	3M4BS	7M	AeP
Germination%	**Control**	96±4.5 ab	94±3.5 ab	95±4.8 ab	92±4.0 ab	94±6.0 ab	91±4.6 ab	100±0.0 a	86±7.5 b	94±5. ab
	**NaCl 100mM**	90±6.5 ab	80±3.5 c	86±3.0 b	76±5.1 cd	93±4.0 ab	90±2.3 ab	96±2.1 a	84±2.4 b	93±2. a
	**NaCl 200mM**	58±5.7 e	55±4.6 e	63±7.2 de	56±3.4 e	70±6.1 d	69±6.8 d	87±6.9 bc	42±7.3 f	78±3. c
	**NaCl 300mM**	36±3.6 f	28.7±3.6 g	38.3±5.0 f	39.6±6.2 f	52±4.8 e	54±4.2 e	67±6.4 de	19±6.1 g	56.7±6 e
Root length(cm)	**Control**	5.5±0.5 ab	5.2±0.6 b	5.1±0.5 b	4.8±0.7 bc	5.9±0.6 ab	5.2±0.6 bc	6.5±0.6 a	4.8±0.6 cd	5.9±0 ab
	**NaCl 100mM**	2.5±0.4 d	2.8±0.4 d	2.7±0.5 d	3.0±0.5 d	4.0±0.6 c	4.2±0.5 c	3.8±0.5 c	2.8±0.4 d	3.7±0 c
	**NaCl 200mM**	1.1±0.3 f	1.4±0.3 f	1.6±0.4 ef	1.4±0.4 ef	1.7±0.3 e	1.6±0.3 e	1.7±0.3 e	0.9±0.3 fg	2.0±0 e
	**NaCl 300mM**	0.3±0.1 h	0.3±0.1 h	0.5±0.2 g	0.4±0.2 gh	0.6±0.2 g	0.5±0.2 g	0.7±0.3 g	0.4±0.2 h	0.6±0 g
Shoot length (cm)	**Control**	2.8±0.4 b	2.7±0.3 bc	2.8±0.3 b	2.3±0.3 cd	3.0±0.4 b	2.7±0.3 bc	3.9±0.4 a	2.8±0.3 bc	3.2±0 ab
	**NaCl 100mM**	1.3±0.3 e	1.0±0.3 e	1.3±0.3 de	1.4±0.3 de	1.7±0.3 d	1.7±0.3 d	1.8±0.3 d	1.4±0.3 de	2.4±0 c
	**NaCl 200mM**	0.3±0.1 g	0.3±0.1 fg	0.2±0.1 g	0.3±0.1 fg	0.4±0.1 f	0.4±0.1 fg	0.5±0.1 f	0.3±0.1 g	0.5±0 f
	**NaCl 300mM**	(h) ranged between 0.1 and 0.25 cm; not compared								
Root weight (mg / plant)	**Control**	31±2.5 ab	30±2.4 b	30±1.8 b	27±2.4 bc	30±2.5 bc	28±1.7 bc	36±2.6 a	27±1.9 c	32±2. ab
	**NaCl 100mM**	16±1.4 e	15±1.1 e	17±1.5 e	15±1.4 e	20±1.5 d	23±1.4 d	25±1.5 c	16±1.4 e	21±1. d
	**NaCl 200mM**	8.2±1.0 g	6.9±1.0 g	6.5±0.9 g	10.6±0.9 f	9.8±0.8 f	10.3±1.0 f	11±1.1 f	8.2±0.8 g	11±1. f
	**NaCl 300mM**	1.2±0.2 i	1.0±0.3 i	1.1±0.2 i	2.0±0.2 h	2.5±0.2 h	2.0±0.2 hi	2.6±0.2 h	1.2±0.2 i	2.4±0 h
Shoot weight (mg / plant)	**Control**	27±2.3 bc	24±2.0 c	25±1.7 c	28±2.4 b	29±1.5 b	28±1.8 b	33±2.6 a	27±1.9 bc	27±1. bc
	**NaCl 100mM**	12±0.9 f	9.5±1.2 f	11±1.5 f	12±1.0 f	14±0.9 e	15±1.1 e	21±1.5 d	12±1.2 f	15±1. e
	**NaCl 200mM**	3.4±0.4 h	3.0±0.4 h	2.8±0.4 h	4.1±0.4 h	4.8±0.6 g	5.1±0.7 g	5.8±0.5 g	3.8±0.5 h	5.4±0 g
	**NaCl 300mM**	(i) ranged between 1 and 3 mg/plant; not compared								

Supplementary Table S3: Results of statistical analysis for relative water content (RWC), SPAD values related to the chlorophyll content of leaves, the CO_2 assimilation rate (Pn), stomatal conductance (gs), transpiration rate (E) and intercellular CO_2 level (Ci) presented in Fig. 4. Different letters indicate significant differences at $p < 0.05$ level using Tukey's *post hoc* test

		RWC	SPAD	Pn	gs	E	Ci
Control	WP	a	b	a	ab	ab	a
	AeP	a	a	a	a	a	a
	1U	a	ab	a	ab	ab	a
	1U/6U	a	ab	a	ab	ab	a
	3U	a	a	a	ab	b	a
	2M	a	bc	a	ab	ab	a
	3M	a	bc	a	ab	ab	a
	3M.4BS	a	bc	a	ab	ab	a
	7M	a	c	a	b	b	a
After salt-treatment	WP	c	d	cd	e	e	b
	AeP	c	bc	b	c	c	bc
	1U	c	d	cd	e	de	b
	1U/6U	c	d	cd	e	de	b
	3U	bc	c	c	e	de	bc
	2M	c	c	b	d	c	bc
	3M	b	c	b	d	d	c
	3M.4BS	b	c	b	d	d	bc
	7M	c	d	d	e	e	b

Supplementary Table S4: Sugar composition of wheat (WP) and *Aegilops* (AeP) parents and addition lines 2M, 3M and 3M.4BS grown in hydroponic solution with and without salt treatment. Data are mean ± standard deviation of five replicates per treatment and the different letters indicate significant differences at the $p < 0.05$ level using Tukey's *post hoc* test.

	Sugars mg g^{-1} FW	WP		AeP		2M		3M		3M.4BS	
		Control	NaCl	Control	NaCl	Control	NaCl	Control	NaCl	Control	NaCl
LEAVES	**Fructose**	1.06 ±0.15 a	0.2 ±0.04 c	0.93 ±0.33 a	0.48 ±0.16 b	0.90 ±0.12 a	0.46 ±0.11 b	0.92 ±0.21 a	0.38 ±0.07a b	1.02 ±0.10a	0.6 ±0.11 b
	**Glucose**	2.81 ±0.54 a	1.64 ±0.26 bc	2.12 ±0.67 ab	1.85 ±0.24 b	2.98 ±0.58 a	2.33 ±0.35 a	2.52 ±0.56 a	2.58 ±0.61 a	2.82 ±0.80 a	1.05 ±0.29 c
	**Galactose**	0 ±0 c	20.22 ±1.61 ab	0 ±0 c	19.31 ±1.61 ab	0 ±0 c	21.01 ±1.44 a	0 ±0 c	20.42 ±1.02 ab	0 ±0 c	17.38 ±1.9 b
	**Sucrose**	11.16 ±1.03 b	14.40 ±0.8 a	10.60 ±1.52 b	16.02 ±1.52 a	10.1 ±1.66 b	13.62 ±0.86 ab	11.91 ±1.62 b	13.82 ±0.74 a	11.32 ±1.71 b	14.88 ±1.21 a
	**Maltose**	0.2 ±0.1 b	0 ±0 c	0.85 ±0.18 a	0 ±0 c	0.6 ±0.10 a	0 ±0 c	0.74 ±0.16 a	0 ±0 c	0.86 ±0.27 a	0 ±0 c
	**S1**	1.68 ±0.23 c	4.3 ±0.734 ab	0.7 ±1.41 c	3.7 ±0.591 b	0.84 ±0.15 a	4.5 ±0.489 ab	1.68 ±0.29 c	4.66 ±0.472 a	1.82 ±0.25 c	3.21 ±0.678 b
	**Raffinose**	0.633 ±0.32 c	2.14 ±0.502 b	1.75 ±0.35 b	3.04 ±0.114 a	2.45 ±0.88 ab	3.02 ±0.378 a	0.87 ±0.37 c	1.96 ±0.572 b	0.92 ±0.37 c	1.98 ±0.478 b
		**WP**		**AeP**		**2M**		**3M**		**3M.4BS**	
		**Control**	**NaCl**	**Control**	**NaCl**	**Control**	**NaCl**	**Control**	**NaCl**	**Control**	**NaCl**
ROOTS	**Fructose**	1.93 ±0.40 d	0.36 ±0.08 f	3.72 ±0.27 b	0.88 ±0.15 e	2.82 ±0.51 c	0.58 ±0.083 a	3.38 ±0.47 bc	0.54 ±0.18 ef	4.42 ±0.27 a	1.54 ±0.55 d
	**Glucose**	1.37 ±0.20 c	0.42 ±0.414 d	2.04 ±0.26 b	0.65 ±0.49 d	1.26 ±0.28 c	0.32 ±0.465 d	2.42 ±0.40 b	0.43 ±0.1 d	3.22 ±0.17 a	1.62 ±0.35 bc
	**Galactose**	0 ±0 c	3.17 ±0.31 b	0 ±0 c	5.26 ±0.54 a	0 ±0 c	3.86 ±0.32 b	0 ±0 c	3.41 ±0.28 b	0 ±0 c	3.90 ±0.41 b
	**Sucrose**	1.56 ±0.56 bc	0.8 ±0.22 cd	2.12 ±0.38 b	1.52 ±0.31 bc	2.62 ±0.18 a	0.9 ±0.38 cd	2.62 ±0.15 a	0.7 ±0.12 d	2.58 ±0.33 a	2.15 ±0.36 b
	**Maltose**	0 ±0 d	1.11 ±0.149 c	0 ±0 d	1.80 ±0.175 b	0 ±0 d	1.66 ±0.152 b	0 ±0 d	2.82 ±0.274 a	0 ±0 d	2.28 ±0.264 a
	**S1**	0.6 ±0.1 c	0.58 ±0.25 c	1.2 ±0.22 ab	1.1 ±0.216 b	1.02 ±0.21 b	0.66 ±0.151 c	1.52 ±0.16 a	0.70 ±0.1 c	0.8 ±0.11 bc	1.28 ±0.203 ab
	**Raffinose**	0.13 ±0.08 c	0.69 ±0.23 ab	0.40 ±0.12 bc	0.9 ±0.35 a	0.44 ±0.12 bc	0.4 ±0.15 bc	0.31 ±0.18 bc	0.32 ±0.05 bc	0.28 ±0.08 c	0.4 ±0.12 bc
